# A pilot study about infertile men’s awareness of their reprotoxic exposures and the intervention of occupational medicine to assess them

**DOI:** 10.1186/s12610-016-0036-5

**Published:** 2016-08-10

**Authors:** Amélie Christiaens, Irène Sari-Minodier, Sophie Tardieu, Oana Ianos, Sébastien Adnot, Blandine Courbiere, Jeanne Perrin

**Affiliations:** 1 CECOS Laboratory of Reproductive Biology, AP-HM La Conception, Pole femmes parents enfants, Marseille, France; 2 Department of Occupational Health, AP-HM La Timone, Marseille, France; 3 Aix Marseille Univ, CNRS, IRD, IMBE, Marseille, France; 4 Department of Public Health, Pole Santé Publique, AP-HM La Conception, Marseille, France; 5 Department of General Practice, Faculty of Medicine - Aix Marseille Univ, 13005 Marseille, France; 6 Department of Reproductive Medicine, AP-HM La Conception, Pole femmes parents enfants, Marseille, France

**Keywords:** Male infertility, Occupational exposures, Semen, Questionnaire, Infertilité masculine, Expositions professionnelles, Sperme, Questionnaire

## Abstract

**Background:**

Male infertility related to professional reprotoxic exposure has been assessed in several studies. Collaboration between occupational physicians and patients can yield information about the preventive measures that can be taken to avoid such exposure. The use of preventive measures is determined by the collaboration between reproductive medicine and occupational medicine and also by the patient’s awareness of reprotoxic occupational exposures. Our andrology laboratory developed a systematic environmental interview that an occupational physician administers before semen analysis to assess patients’ occupational reprotoxic chemical and physical exposures. This observational prospective study evaluated patients’ feelings regarding this interview. The main outcome measure was the participants’ score to determine their general reprotoxicant knowledge. The study also evaluated the patients’ satisfaction about the interview with occupational physician and their attitude about reproductive toxicants.

**Results:**

The mean score for general knowledge of reprotoxicants was 9.6 ± 2.7/16. The most frequently underestimated reprotoxic factor was excessive heat (34.7 % correct responses). In cases of semen parameter abnormalities AND recognized occupational reprotoxic exposure, 63.2 % of the patients said they would use individual protective devices, and 55.1 % said they would temporarily adapt their workstation. Regarding the interview with the laboratory’s occupational physician, 80.7 % considered it moderately or very useful. Of the interviewed patients, 46.2 % reported having changed their living habits 2 months after the interview, and 88.5 % were satisfied or very satisfied with the care they received. All of the respondents said it would be useful to extend the interview to include their wives.

**Conclusions:**

The data suggest that patients’ knowledge about reprotoxic exposures can be improved, particularly knowledge related to physical exposure. The vast majority of patients were satisfied with the introduction of this new collaboration between reproductive and occupational medicine.

## Résumé

**Contexte:**

Plusieurs études ont mis en évidence des infertilités masculines en lien avec une exposition reprotoxique professionnelle. L’interaction entre médecin du travail et patient peut faire émerger des informations sur les mesures preventives à mettre en place pour éviter ces expositions. L’utilisation de ces mesures préventives est déterminée par la collaboration entre médecin de la reproduction et médecin du travail et également par la conscience du patient de subir une exposition professionnelle reprotoxique. Notre laboratoire de spermiologie a mis en place un entretien systématique environnemental, assuré par un médecin du travail avant l’analyse de sperme, dans le but d’établir les expositions professionnelles reprotoxiques chimiques et physiques de chaque patient. Cette étude observationnelle prospective évalue la perception des patients de cet entretien. Le critère d’évaluation principal est le score de chaque patient évaluant ses connaissances générales sur les reprotoxiques. L’étude évalue également la satisfaction des patients à propos de cet entretien avec le médecin du travail et leur attitude vis à vis des reprotoxiques.

**Resultats:**

Le score moyen de connaissances générales sur les reprotoxiques était de 9.6 ± 2.7/16. Le facteur reprotoxique le plus fréquemment sous estimé était la chaleur excessive (34.7 % de bonnes réponses). En cas d’altération des paramètres spermatiques associée à des expositions professionnelles reprotoxiques, 63.2 % des patients se disaient prêts à utiliser des dispositifs de protection individuelle, et 55.1 % à accepter une adaptation temporaire de leur poste de travail. Concernant l’entretien avec le médecin du travail du laboratoire de spermiologie, 80.7 % le considéraient modérément ou très utile. Parmi les participants, 46.2 % rapportaient un changement dans leurs habitudes de vie 2 mois après l’entretien, et 88.5 % étaient satisfaits ou très satisfaits des soins reçus. Tous les participants trouveraient utile d’étendre cet entretien à leur compagne.

**Conclusions:**

Nos résultats suggèrent que les connaissances des patients sur les expositions reprotoxiques peuvent être améliorées, particulièrement en ce qui concerne les expositions physiques. La grande majorité des patients était satisfaite de l’introduction de cette nouvelle collaboration entre médecine de la reproduction et médecine du travail.

## Background

Male infertility related to professional reprotoxic exposure has been assessed in several studies, many of which are case–control studies or standardized interviews. Nevertheless, patients interviewed in studies about occupational exposures may not be aware of their reprotoxic exposure [1–3]. According to a recent systematic review, the main occupations that are significantly associated with semen parameters impairment are workmen, painters, farmers, welders, plumbers and technicians; occupations that involve exposure to solvents, heavy metals, heat, vibrations and ionizing radiations are also associated with semen impairment [4, 5].

Collaboration between occupational physicians and patients can yield information about the preventive measures that can be taken to avoid such exposure. The use of preventive measures is determined by the patient’s awareness of reprotoxic occupational exposures and by the collaboration between reproductive medicine and occupational medicine. Our andrology laboratory developed a systematic environmental interview that an occupational physician administers before semen analysis to assess patients’ reprotoxic exposure [3].

The objective of our study is to evaluate patients’ feelings about this new occupational reprotoxic exposure interview and to evaluate their awareness of professional and environmental reprotoxic risks.

## Methods

### Study population

In this observational prospective study, patients were recruited from the population of male patients seen during routine diagnosis in andrology laboratory of the fertility clinic. Every patient that visited the laboratory was interviewed by the occupational physician before providing a semen sample the same day or within 3 days if a same-day sample could not be provided.

The inclusion criteria were any male patient from 18 to 55 years old who had a good command of the French language, visiting the laboratory for the first time and volunteered to participate.

The exclusion criteria were as follows:

- Any identifiable cause of infertility, such as bilateral cryptorchidism, bilateral testicular hypotrophy, bilateral varicocele, congenital absence of the vas deferens, endocrine or central hypogonadism, a history of chemotherapy, genetic or chromosomal abnormalities, or exposure to diethylstilbestrol in utero. These patients were excluded because their medical history could induce an alteration of their general knowledge about fertility.
- Current use of any medication known to impair semen parameters, such as cardiotropics, anti-epileptics, or psychotropics (see full list in *De Fleurian* et al.’s article [3]).

### Study design and setting

The study consisted of a survey designed to assess patients’ knowledge about reproductive toxicants and patients’ satisfaction with this new collaborative effort involving reproduction and occupational medicine.

All of the subjects were recruited between February and April 2015 in the Reproductive Medicine Laboratory, La Conception University Hospital, Marseille, France.

### Survey

#### Questionnaire contents

Two self-administered written questionnaires were designed to be completed with a time interval of 2 months (see full text of the questionnaires in Appendix).

The first questionnaire was administered before the first semen analysis. It evaluated 1) the patients’ perceptions of the potential impact of occupational and environmental habits on fertility, and 2) their feelings about the interview with the occupational physician.

The second questionnaire was administered 2 months after patients met the occupational physician and obtained the results of semen analysis. It assessed the potential changes the patients made in response to the previous encounter.

#### Questionnaire administration

This survey was conducted in a clinical setting. It was based on voluntary participation and did not affect patient care. Approval from the Aix-Marseille University Ethics Committee was obtained (case number 2015-0603004). The study was also registered with the local representative of the French Data Protection Authority at the Hospital.

Approximately 30 min before the appointment with the occupational physician, the patients were given written information about the study and the contents of the questionnaire. They were also asked to sign a written consent form and provide their e-mail address to be contacted to complete the second self-response questionnaire. The patients were then given the first self-administered questionnaire, which took approximately 10 min to complete. They then met with the occupational physician and were asked to provide a semen sample.

Two months later (the necessary estimated time to allow patients to receive their semen sample analysis results and possibly change their habits regarding professional and private reprotoxic exposure), they received the second self-administered questionnaire in an e-mail. It took approximately 5 min to complete.

#### Interview with occupational physician

The interview with occupational physician set place after the first self-administered questionnaire, before the semen collection. All the interviews in our study were achieved by the same occupational physician. The interview was based on a questionnaire designed to collect information about occupational exposures to physical and chemical reprotoxic factors. Environmental exposures and medical history were also assessed as confounders. The full questionnaire and the contents of the interview are described in detail in De Fleurian et al.’s previous article on the topic [3].

Briefly, the occupational physician’s questionnaire was divided into several parts:

- Part 1 assessed general patient information, such as age and body mass index.
- Part 2 assessed the confounding factors known to impair semen parameters.
- Part 3 assessed suspected environmental confounders, such as leisure activities involving toxicants (tobacco smoke; alcohol use and drugs), chemicals or physical risk.
- Part 4 asked patients about their potential occupational reprotoxic exposures using 3 parameters: 1) social and economic class, as defined by the French National Institute of Statistics and Economic Studies [6]; 2) present or past occupational activities; and 3) occupational exposure to reprotoxic chemical or physical factors specific to certain professional activities.

## Results

### Population

**Fig. 1:**
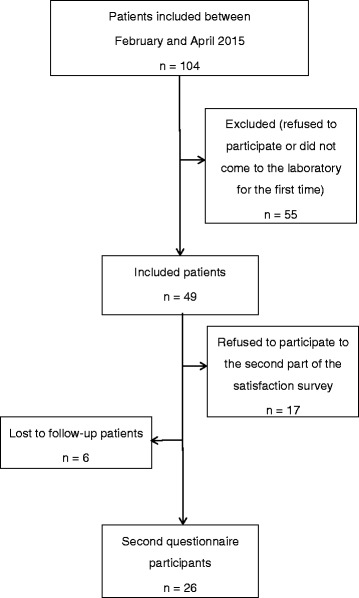
Flow chart of the survey

**Fig. 2:**
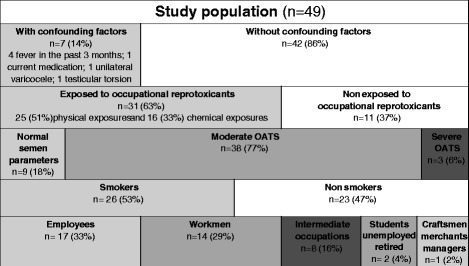
Characteristics of the population. “Employees” include Public service employees, clerical jobs, service and sales workers; « Workmen » include skilled or unskilled workers, agricultural labourers; « Intermediate Occupations » include foremen, technicians and associate professionals from health, teaching, public service… (function between managers and operating agents)

Forty-nine patients were included and answered the first questionnaire (Fig. 1). Their mean age was 36 ± 6.4 years, and their mean body mass index was 25.2 ± 4.2 kg.m^-2^. The characteristics of the population are presented in Fig. 2.

### Evaluation of patients knowledge and attitude about reproductive toxicants

The mean score for general knowledge was 9.6 ± 2.7/16. The most frequently unrecognized reprotoxic factor was excessive heat (35 % answered correctly). Regarding prior knowledge of reprotoxic exposure, 26 % patients (13/49) reported that they were already aware of reprotoxic exposures as a result of billboards (*n* = 6) or medical consultation (*n* = 6).

The evaluation of patients’ self-perceived occupational exposures and the concordance with exposures assessed by the interview with the occupational physician are presented in Table 1.

**Table 1:** Evaluation of patients’ self-perceived occupational exposures and concordance with exposures assessed by the occupational physician

Questions	Answers		
Self-perceived reprotoxic exposure (3 groups)	Yes *n* = 8 (16 %) 2 policemen; 1 fireman; 1 dentist; 1 bus driver; 1 barman; 1 cook; 1 bricklayer.	I don’t know *n* = 7 (15 %) 2 administrative employees; 1 technician; 1 student; 1 member of the army; 1 merchant; 1 artist.	No *n* = 34 (69 %)
Reprotoxic exposure assessed by the interview with occupational physician in each group	*n* = 8 - 5 physical occupational exposures (4 excessive heat; 2 vibrations; 1 ionizing radiations). - 7 chemical occupational exposures (3 PAH; 2 gas & fumes; 2 passive tobacco smoke; 2 solvents; 1 heavy metals; 1 colorants; 1 atmospheric pollution).	*n* = 4 5 physical occupational exposures (3 excessive heat; 1 vibrations).	*n* = 19 - 16 physical occupational exposures (12 excessive heat; 5 vibrations) - 9 chemical occupational exposures (6 gas & fumes; 5 PAHs; 4 solvents; 2 colorants; 2 cement; 1 pesticides; 1 heavy metals).
Concordance between self-perceived and assessed reprotoxic exposure in each group	8/8 = 100 %	-	15/34 = 44 %

In total, 8 out of 31 (26 %) exposed patients were aware of their reprotoxic exposure.

The patients reported that if they had semen parameters abnormalities AND recognized occupational reprotoxic exposure, 63 % (*n* = 31) would adopt individual protection devices, 55 % (*n* = 27) would temporarily change their workstation. Eight percent (*n* = 4) would not make any changes, and 92 % (*n* = 45) would change their living habits.

### Evaluation of patients satisfaction about the interview with occupational physician

**Fig. 3:**
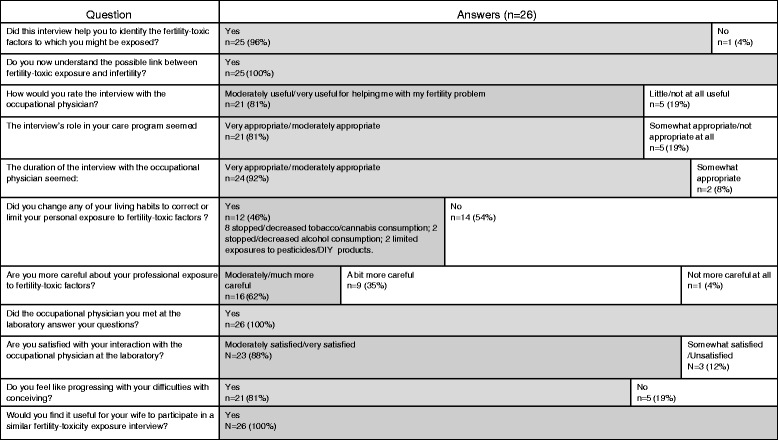
Answers to the second questionnaire

Twenty-six patients answered the second questionnaire; answers are presented in Fig. 3.

## Discussion

This preliminary study evaluated patients’ satisfaction with an interview with an occupational physician that took place during the first semen analysis of infertile males. We also assessed the general knowledge of these patients about reprotoxic exposures.

### Level of reprotoxic knowledge

The mean score for general knowledge of reprotoxicants shows that patients are quite aware of the association of infertility and risk factors. There are very few articles in the literature about this subject. In *Hussain T*. et al.’s [7] awareness evaluation of the general population based one-on-one interviews, correct responses were limited (for example, 43 % and 48 % of males considered mumps and smoking, respectively, as culprits). *Remes O*. et al.’s [8] semi-structured interviews showed that students had a superficial understanding of the environmental risks associated with environmental contaminants, sexually transmitted diseases and lifestyle and that at times, they relied on media reports and anecdotal information to support their beliefs. The researchers concluded that patients exhibited a general understanding of environmental risks associated with infertility but that young adults are overly optimistic that healthy lifestyle behaviors will safeguard their future fertility. No awareness or knowledge scores were found in the literature.

We observed that excessive heat was underestimated as a reprotoxic risk factor, although it was the most frequently reported reprotoxic exposure in our study and is a recognized reprotoxic factor [2, 9, 10]. Despite the widespread nature of this occupational reprotoxic exposure, patients do not take it as seriously as they should. Several studies have examined the prevention of heat-related illnesses among outdoor workers in warm countries [11, 12], farmers [13], and bus drivers [1] and have managed to reduce heat-related morbidity. All of these studies examined all types of heat-related illness (and not specifically fertility impairment), but they indicate a trend toward developing heat-related regulations or guidelines to minimize the risk of occupational heat infertility in affected men. Concentrating efforts on increasing awareness of heat exposure could be an efficient prevention strategy for preserving men’s reproductive health.

Moreover, a comparison of the responses indicating that patients thought they had an occupational reprotoxic exposure before the occupational physician’s interview and having an exposure registered by the occupational physician shows that 1) patients who are aware of the risk of exposure are correct; 2) approximately 2 out of 3 unaware patients underestimate their exposure to both physical and chemical reprotoxic factors; 3) no patient overestimated his exposures.

### Patients’ sources of information about reprotoxicants

According to our satisfaction survey, the patients learned about reprotoxic exposure risks through billboard campaigns and from a not dedicated medical consultation. No specific study has assessed the best way for a reprotoxic prevention campaign to be efficient, but several campaigns have successfully increased workers’ awareness of occupational risks: *Quach T*. et al. trained managers and owners of nail salons to reduce exposure to solvents [14]; *Malchaire J*. et al. trained small groups of workers using a 4-step program [15]; and *Riedel JE*. et al. described an example of a new “health and productivity dashboard” [16]. Such information sources could be applied to reprotoxic exposure prevention in the future.

### The preventive role of occupational medicine

We observed that a minority of patients (1/30) received information through their company’s occupational physician, despite having seen him/her in the past 6 months (19/47). This finding suggests that discussing reprotoxicants with patients is not a priority of such consultations, even when the patients are of reproductive age.

Patients’ rights regarding chemical exposures are clearly addressed in the labor laws, but little is mentioned about physical exposure. Additionally, the European REACH regulations address the effects of chemical toxicants on reproduction and development. Nevertheless, our results demonstrated that physical reprotoxic exposures are very common, and to our knowledge, no regulations are available for physical reprotoxic exposure (except for ionizing radiations). This could also explain the lack of information and preventive efforts provided by occupational physicians.

The patients’ poor self-assessments of their reprotoxic exposures during the occupational consultation could also be related to another important factor that was assessed in previous studies: patients may avoid talking about personal health problems at work even if the occupational physician could improve them, because the patients are 1) ignorant of the role and skills of occupational physicians; 2) doubtful of the occupational physician’s competence; and 3) uncertain about the occupational physician’s independence from their employer and do not want to have their intimate health matters revealed to the company [17].

In our study, 6 out of 49 patients reported having received information during a medical consultation, likely from a specialist other than their occupational physician. In our population, we can estimate that at least 10 % (5/49) of the patients did not have any link to occupational medicine because they were not salaried workers (1 secondary school teacher, 1 student, 1 dentist and 2 unemployed patients). We suggest that general practitioners should be in a position to inform such patients. Although many studies show that professional collaboration between occupational physicians and general practitioners is difficult [18, 19], other studies also show that there are no significant factors that encourage patients to choose between their general practitioner or their occupational physician regarding health matters at work [20].

### Impact of the reprotoxic exposure analysis on patients

In their responses to the first questionnaire of the survey, a majority of the patients declared being willing to change their individual (92 %) and professional (63 %) behaviors regarding reprotoxic exposures; only 8 % of the patients would not make any changes. In their responses to the second questionnaire (2 months later), 38 % of the patients reported having changed their professional habits, and 46 % reported having changed their personal habits. These results could suggest 1) sufficient pre-existing use of preventive measures (10/49 patients declared in the first questionnaire that they had access to protection devices, and 9/49 reported that they used them all the time or frequently); 2) a pre-existing change in patients’ behaviors in response to their hope to achieve pregnancy without waiting for advice from the occupational physician; and 3) an unconscious resistance to behavior changes [21].

### Indications and perspectives for reproductive health

The routine collaboration between reproductive and occupational medicine could improve the management of infertile men by allowing a better detection, characterization and eviction of reprotoxic exposures by using the specific skills of occupational physician in a separate interview. It could also increase the patient’s perception of the reprotoxic exposures he faces in his domestic and occupational environments, allowing a more active behavior to protect himself from these reprotoxicants. Moreover, occupational physicians are qualified to help employees and employers to insure a safer working environment, which could benefit to infertile patients but also to other young employees willing to conceive. We hypothesize that the setup of this collaboration at the beginning of the patient’s Assisted Reproductive Therapy path could improve the patient’s reproductive and general health, which is an important issue when building a family.

### Limitations

Limitations of our study are: a) the size of our population, which might not confer adequate statistical power; b) the rate of patients who did not want to participate to the second part of the satisfaction survey or were lost of follow-up (47 % of included patients), which may be due to the relatively long delay between the two questionnaires (two months); c) the exclusion of patients without a good command of the French language or who did not want to participate, which could alter the estimation of the general knowledge about reprotoxicants in the population of infertile men.

## Conclusion

The vast majority of our patients were satisfied with the introduction of a new interview with an occupational physician, primarily because the interviews resulted in 1) increased awareness of their own private and occupational reprotoxic exposure; and 2) insight into behavior changes that could directly improve their reproductive health.

Our results also suggest that patients’ knowledge about reprotoxic exposure can be improved, particularly in regard to physical exposure.

This preliminary study encourages us to develop the collaboration between occupational medicine and reproductive medicine by:

- Providing better information to companies’ occupational physicians and occupational health professionals (including nurses);
- Inviting collaboration between reproductive medicine professionals and company’s occupational physicians or general practitioners to establish corrective measures in cases recognized associations between semen parameter abnormalities and occupational reprotoxic exposure;
- Initiating a similar program for female infertility prevention.

## Abbreviations

CREER, couple reproduction enfant, environnement et risques; OATS, oligo-astheno-terato-zoospermia; PAHs, polycyclic aromatic hydrocarbons; REACH, registration, evaluation, authorisation and restriction of chemicals; WHO, World Health Organization

## Appendix

Semen sample analysis

The patients provided ejaculate by masturbating into a sterile container after a 3- to 6-day period of sexual abstinence. The semen was analyzed after 30 min liquefaction at 37 °C in accordance with the current (2010) WHO guidelines [22] for all parameters except morphology. Indeed, sperm morphological anomalies were described using *David el al*.’s modified classification [23]. This classification describes normal morphology, 7 head abnormalities, 3 midpiece abnormalities and 5 tail abnormalities. The percentages of morphologically normal and abnormal spermatozoa were estimated based on 100 spermatozoa viewed at ×1000 magnification.

Normal semen parameters were defined according to the following criteria: total sperm count over 39 million/ejaculate AND concentration over 15 million.mL^-1^ AND percentage of sperm with progressive motility over 32 % [22] AND percentage of sperm with normal morphology (typical forms) over 15 % [23]. Abnormal semen parameters were separated in two types [7]:

- Severe oligo-astheno-terato-zoospermia (OATS) [22, 24]: total sperm count below 39 million/ejaculate AND concentration below 5 million.mL^-1^ AND progressive motility below 32 % AND typical forms below 15 %.
- Moderate OATS: other below-normal values for these parameters.

First patient questionnaire

General awareness of reprotoxic exposure:

1. In your opinion, which of these environmental factors are known to be fertility-toxic? Choose one or more answers.

Heavy metals (lead, mercury, cadmium…) (reprotoxic)

Solvents (paint, glue, varnish, ink…) (reprotoxic)

Hydrocarbons (oil derivatives, asphalt, gasoline fumes…) (reprotoxic)

Gas and fumes (welding, plastics, plant fumes…) (reprotoxic)

Pesticides (insecticides, phytosanitary products…) (reprotoxic)

X-rays (ionizing radiation) (reprotoxic)

Excessive heat (reprotoxic)

Cement (reprotoxic)

Alcohol (reprotoxic)

Tobacco (reprotoxic)

Vibrations (reprotoxic)

Carbonated drinks (not reprotoxic)

Anabolic steroid food supplements (reprotoxic)

Mineral water (not reprotoxic)

Cannabis (reprotoxic)

Ecstasy (not reprotoxic)

Self-perceived reprotoxic exposure:

2) In the last 6 months, do you think you have been exposed to fertility-toxic factors IN YOUR PERSONAL ENVIRONMENT? Yes No I don’t know

If yes, cite them:

3) In the last 6 months, do you think you have been exposed to fertility-toxic factors IN YOUR PROFESSIONAL ENVIRONMENT? Yes No I don’t know

If yes, cite them:

4) Do protective measures against fertility-toxic exposure exist at your workplace? Yes No I don’t know

If yes, cite them:

5) If yes, how often do you use them? All the time Often Rarely Never
6) Have you been informed about the existence of fertility-toxic exposure? Yes No

If yes, tell us how: Select all that apply

Billboards

Specific consultation with occupational medicine professionals

During a consultation with another medical professional for another reason (for example, during a visit with your general practitioner)

Other: specify

Relationship with occupational medicine:

7) Have you ever met an occupational physician in your current job (or in the past 6 months if you recently changed jobs)? Yes No (if no, go directly to question 9)
8) Have you ever discussed your fertility-toxic exposure risk with your company’s occupational physician? Yes No

Protective measures:

9) Assuming that you had severe semen parameter impairments AND recognized fertility-toxic exposures IN YOUR OCCUPATIONAL ENVIRONMENT, would you be willing to: (choose all that apply)

Adopt individual protection measures (ex: using an extractor fan, wearing gloves)

Accept a temporary workstation change (ex: spend less time sitting or standing)

Accept a temporary change of jobs (ex: temporarily leave your current job for another one that would not expose you to fertility-toxic factors)

None of these proposals

10) Assuming that you had severe semen parameter impairments AND recognized fertility-toxic exposures IN YOUR PERSONNAL ENVIRONMENT, would you be willing to change your lifestyle habits (ex: stop smoking tobacco, discontinue specific leisure activities)? Yes No

Now you will meet with our laboratory’s occupational physician for a more detailed consultation about the fertility-toxic exposures you might encounter.

11) Do you have any questions you wish you could ask a doctor about your fertility disorder? Yes No If yes, cite them:

Second patient questionnaire

General interest in the interview with the occupational physician:

1. Did this interview help you to identify the fertility-toxic factors to which you might be exposed? Yes No
2. Do you now understand the possible link between fertility-toxic exposure and infertility? Yes No
3. How would you rate the interview with the occupational physician? Very useful for helping me with my fertility problem Moderately useful Less useful Not at all useful

Behavior changes:

4) Did you change any of your living habits to correct or limit your personal exposure to fertility-toxic factors? Yes No If yes, cite the changes:
5) Are you more careful about your professional exposure to fertility-toxic factors? A lot more careful Somewhat more careful No more careful than before

Practical organization of the interview:

6) The duration of the interview with the occupational physician seemed:

Very appropriate Moderately appropriate Somewhat appropriate Not appropriate at all

7) The interview’s role in your care program seemed:

Very appropriate Moderately appropriate Somewhat appropriate Not appropriate at all

Globally:

8) Did the occupational physician you met at the laboratory answer your questions? Yes No
9) Are you satisfied with your interaction with the occupational physician at the laboratory? Very satisfied Moderately satisfied Somewhat satisfied Unsatisfied
10) Do you feel like progressing with your difficulties with conceiving? Yes No
11) Would you find it useful for your wife to participate in a similar fertility-toxicity exposure interview? Yes No
